# Deep Learning-Based Artificial Intelligence System for Automatic Assessment of Glomerular Pathological Findings in Lupus Nephritis

**DOI:** 10.3390/diagnostics11111983

**Published:** 2021-10-26

**Authors:** Zhaohui Zheng, Xiangsen Zhang, Jin Ding, Dingwen Zhang, Jihong Cui, Xianghui Fu, Junwei Han, Ping Zhu

**Affiliations:** 1 Department of Clinical Immunology, Xijing Hospital, Fourth Military Medical University, Xi’an 710032, China; zhengzh@fmmu.edu.cn (Z.Z.); dingjin@fmmu.edu.cn (J.D.); fuxianghui0225@126.com (X.F.); 2 School of Automation, Northwestern Polytechnical University, Xi’an 710072, China; xszhang116@gmail.com (X.Z.); zhangdingwen2006yyy@gmail.com (D.Z.); 3 Lab of Tissue Engineering, College of Life Sciences, Northwest University, Xi’an 710069, China; cjh@nwu.edu.cn

**Keywords:** lupus nephritis, renal biopsy, histopathology, deep learning, artificial intelligence

## Abstract

Accurate assessment of renal histopathology is crucial for the clinical management of patients with lupus nephritis (LN). However, the current classification system has poor interpathologist agreement. This paper proposes a deep convolutional neural network (CNN)-based system that detects and classifies glomerular pathological findings in LN. A dataset of 349 renal biopsy whole-slide images (WSIs) (163 patients with LN, periodic acid-Schiff stain, 3906 glomeruli) annotated by three expert nephropathologists was used. The CNN models YOLOv4 and VGG16 were employed to localise the glomeruli and classify glomerular lesions (slight/severe impairments or sclerotic lesions). An additional 321 unannotated WSIs from 161 patients were used for performance evaluation at the per-patient kidney level. The proposed model achieved an accuracy of 0.951 and Cohen’s kappa of 0.932 (95% CI 0.915–0.949) for the entire test set for classifying the glomerular lesions. For multiclass detection at the glomerular level, the mean average precision of the CNN was 0.807, with ‘slight’ and ‘severe’ glomerular lesions being easily identified (F1: 0.924 and 0.952, respectively). At the per-patient kidney level, the model achieved a high agreement with nephropathologist (linear weighted kappa: 0.855, 95% CI: 0.795–0.916, *p* < 0.001; quadratic weighted kappa: 0.906, 95% CI: 0.873–0.938, *p* < 0.001). The results suggest that deep learning is a feasible assistive tool for the objective and automatic assessment of pathological LN lesions.

## 1. Introduction

Lupus nephritis (LN) affects up to 40% of adults and 80% of children with systemic lupus erythematosus and is a major cause of morbidity and mortality [1]. In China, nephropathy is present in 47.4% of patients with systemic lupus erythematosus [2], and LN accounts for 54.3% of all secondary glomerular diseases [3].

Accurate renal impairment evaluation is vital for guiding treatment and improving the prognosis of LN [4,5] and to date, renal biopsy is the gold standard for LN diagnosis. The International Society of Nephrology and the Renal Pathology Society (ISN/RPS) categorised glomerular lesions in LN from class I to VI and the activity of renal involvement in terms of active and chronic indices [6,7]. There is also a well-recognised association that links histopathological LN findings to the clinical course, with mesangial nephritis (class II) carrying the best renal prognosis, whereas proliferative nephritis (classes III and IV) presents with a more aggressive course [8,9].

Although the current ISN/RPS classification system has been recognised and adopted by renal pathologists worldwide, obstacles to its application remain, in that it is highly subjective and depends on the experience of the pathologist. Wilhelmus et al. showed that the global interobserver agreement regarding the recognition of class III and IV lesions was very poor, and highly experienced pathologists had higher agreement than less experienced pathologists [10]. Recently, a systemic review by Dasari et al. indicated that the interpathologist agreement in assessing the LN ISN/RPS class, active indices, and chronic indices was poor to moderate overall [11].

To improve this situation and assess LN renal pathology more accurately, efforts such as performing nephropathologist training, continually updating the pathology assessment guidelines, and using objectively measurable biomarkers are warranted. In addition, widespread use of digital pathology and advances in artificial intelligence (AI) [12] have significant potential to aid histopathology diagnostics, offering novel methods to improve accuracy, reproducibility, speed, and to ease the workload of pathologists [13]. At the same time, it enabled the application of machine or deep learning algorithms to facilitate more accurate LN diagnosis [11,14]. These AI approaches have an accuracy similar to that of expert pathologists and, more importantly, improve human reader performance when used together with standard protocols in detection and diagnostic scenarios [12,15,16,17].

To the authors’ knowledge, this is the first report on AI-driven pathological human LN diagnosis using whole-slide images. Previous studies have focused on other renal diseases or used mice biopsies. In this study, a deep learning model was trained and validated using human kidney biopsy whole-slide images (WSIs) stained with periodic acid-Schiff (PAS). The aim was to produce a detection system to identify glomeruli and classify glomerular lesions automatically from LN patients, which would help improve diagnostic accuracy and facilitate effective renal histopathology assessment.

## 2. Materials and Methods

### 2.1. Datasets/Specimen Preparation

Retrospective analysis of renal biopsy findings was performed in patients at Xijing Hospital between 2011 and 2019. A total of 349 slides of biopsy WSIs from 163 patients were employed for subsequent annotation and detection. An additional 321 unannotated biopsy WSIs from 161 patients were used for performance evaluation at the kidney level.

These renal samples were obtained by needle biopsy, and the tissues were processed using standard light microscopy techniques. Specimens stained by PAS were employed because PAS-stained kidney WSIs can yield the best concordance between pathologists and deep learning segmentation across all structures [18,19]. Several demographic and clinical features were collected from these patients at the time of biopsy. Digital images were created using a Unic digital scanner (Precise 500B, Unic Technologies Inc. Beijing, China) that has a resolution of 0.12 μm/pixel at 40× objective. Digital images were saved in JPEG format at 24-bit depth and a horizontal and vertical resolution of 96 dpi.

### 2.2. Annotation Procedure

Using a modified version of the one given by LabelImg [20], a free and open-source graphic image annotation tool, three experienced nephropathologists annotated and recorded the glomerulus positions and coordinates in all WSIs (Figure S1). The pathological findings in all glomeruli were evaluated and annotated as ‘slight’, ‘severe’, ‘sclerotic’, and ‘incomplete’. In class II LNs, almost all glomeruli were annotated as ‘slight’, whereas in classes III and IV, the percentages of glomeruli categorised as ‘severe’ were <50% and >50%, respectively. Incomplete glomerulus may have a low diagnostic value; however, it can easily interfere with other categories and induce false detections. Therefore, it was listed as a separate category. Glomeruli contours were annotated to generate a mask for each image. In addition, the image quality was evaluated, and poor-quality images that were not suitable for subsequent evaluation were discarded, such as those showing tissue slices that were collapsed due to external forces, not in focus, or obscured by dust. Three nephropathologists with over 15 years of experience reviewed the cropped images. Each image was firstly independently reviewed by two experts. If they agreed, then this image was selected for further model training, whereas if they disagreed, a third review was requested, and the majority opinion among the three experts was considered as final. A total of 1312 severe, 1617 slight, 449 sclerotic, and 528 incomplete glomeruli were labelled in 349 separate images.

### 2.3. Image Preparation and Module Design

This experiment consisted of two modules: localisation and classification. In both modules, histogram matching [21] was used for colour normalisation of the image as the first step. Colour normalisation can reduce staining-induced colour differences.

The localisation dataset was obtained by downsampling WSIs, aiming to reduce the image resolution to obtain a thumbnail image. This approach improves the positioning speed and reduces the time and space required. The classification dataset was generated by data interception based on WSIs. To improve the algorithm robustness against variations in tissue morphology and staining, spatial (flipping, rotation, zoom, translation) and colour (Gaussian noise, hue shifting) augmentation techniques were used for data enhancement. A dataset with 1047 kidney tissue images was finally generated for the localisation module and split into training (*n* = 973), validation (*n* = 37), and test (*n* = 37) datasets. After data interception and enhancement, 12,113 glomerular images (including non-glomerular areas) were obtained for glomerular classification (training: 9688; validation: 1212; testing: 1213).

**Figure 1:**
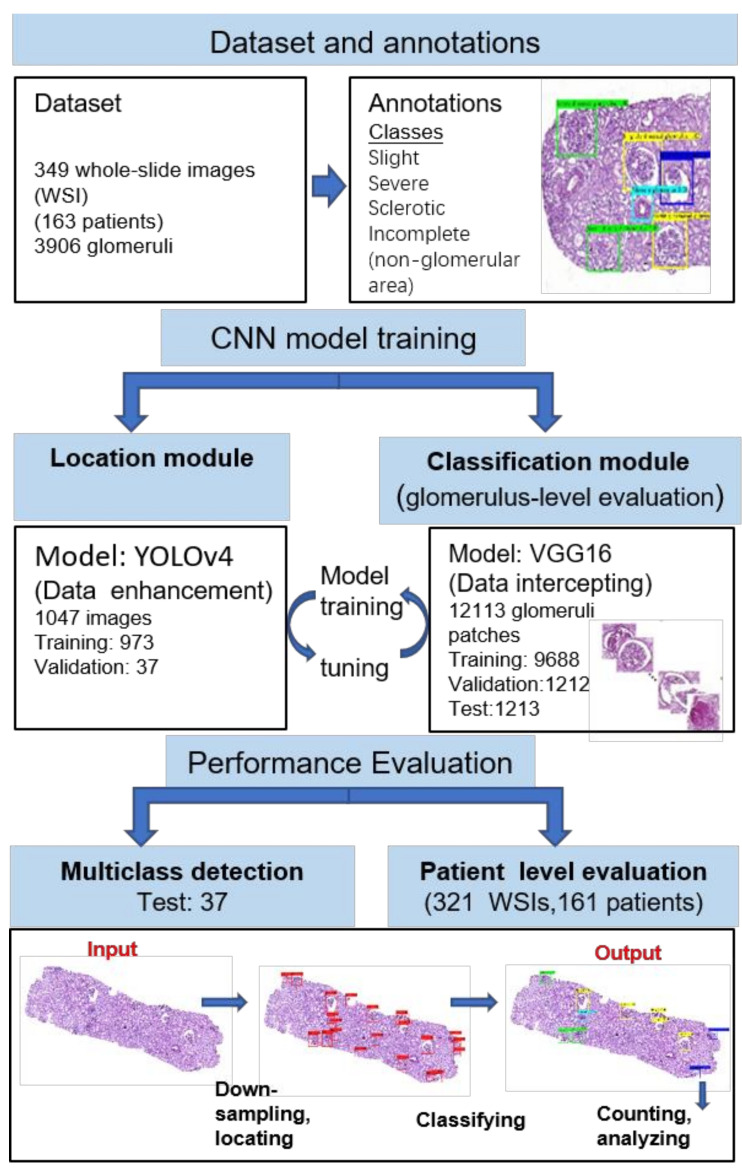
Overview of the experimental design. Given an input image, the glomeruli are located by using the localisation module, with subsequent analysis using the classification module that identifies the lesion class of each glomerulus.

To improve the detection efficiency and reduce the data processing time, glomerular localisation was obtained in the low-resolution images and localised high-resolution images were used to classify glomerular lesions. Figure 1 shows the workflow. Given an input WSI, the high-resolution image was first downsampled. Then, the obtained low-resolution image was input into the localisation module, which generated coarse glomerular localisations as its output. Next, based on the coarse glomerular localisations, high-resolution glomerular patches were cut out from the high-resolution WSIs and used as the input for the classification module.

### 2.4. Glomerular Detection and Localisation

Considering both detection accuracy and time, YOLOv4 [22] was selected as the model for the localisation module (Figure S2). This model could not be directly trained because of significant differences in the image heights and widths. Therefore, the size of each input image was scaled and the training parameter anchors were set before training. To acquire proper anchors, the ground-truth data were downsampled to 256 × 768 pixels and the k-means algorithm was used to cluster the widths and heights of the ground-truth bounding boxes to set the anchors. Subsequently, a YOLOv4 was trained for 50 epochs, at 209 iterations per epoch, with batch sizes of four images (256 × 768 pixels).

### 2.5. Classification of Glomerular Findings

In the classification module, the convolutional neural network (CNN) model VGG16 [23] was chosen, which performed better than the other CNN models, such as GoogLeNet (accuracy: 71.60%) and ResNet (accuracy: 76.54%), in the preliminary experiments. To extract the features of a glomerulus, the VGG16 model was trained for 80 epochs with a batch size of four. In the training process the learning rate was reduced by 10 times per 10 epochs (Figure S3). After layer-by-layer extraction of the CNN, the feature vector finally obtained contained various information such as structural distance, morphologic, colour, and textural information. Stochastic gradient descent was used as the optimisation algorithm, and categorical cross-entropy was employed as the associated loss function for training. In this module, the input glomeruli were mainly divided into five categories: ‘non-glomerular areas’, ‘slight’, ‘severe’, ‘sclerotic’, and ‘incomplete’. Typically, if the glomerulus had the highest probability of falling into a certain category, it fell into that category. However, based on observation, the degree of prediction was sometimes close to a critical state where the probability of being the most likely category was approximately the same as that of the second most likely category. To this end, a new category was added: ‘uncertain glomerulus’. The threshold value was set to 0.05, which meant that if the difference between the probability of being the most likely category and the probability of being the second most likely category was less than the threshold, the glomerulus was judged as an ‘uncertain glomerulus’.

### 2.6. Multiclass Glomerular Performance Evaluation at the Glomerular Level

Integrating the localisation and classification modules, high-resolution WSIs was provided as the model input. After colour normalisation, downsampling, and localisation, high-resolution glomerular images were obtained and classified. The classification module was slightly different from that described in the previous section, which filtered out ‘non-glomerular area’ predictions. To improve fault tolerance, the ‘uncertain glomerulus’ category was also used, which represented the extent of a lesion that was not readily distinguishable and needed to be diagnosed by a pathologist.

### 2.7. Performance Evaluation of LN Classification among Nephrologists and the AI Model at the Renal Level

Once all glomeruli had been classified, the method of generating manual kidney-level predictions of classification depended on a simple majority count. Simply put, if all glomerular lesions were classified as ‘slight’, the patient was categorised as class II. If <50% or >50% of the glomeruli were classified as ‘severe’, the patient was categorised as class III or IV, respectively. LN diagnosis was confirmed by three experienced nephropathologists, which was considered the ground truth.

Before the evaluation, the images in the annotated group were pre-evaluated, and there were few mistakenly detected glomeruli that could lead to classifications different from those of the nephrologists. According to the nephrologists, most cases that had only one ‘severe’ glomerulus were included in class II, whereas those with two ‘severe’ glomeruli were mostly categorised as class III. For most kidneys in which the number of ‘sclerotic’ glomeruli was not greater than the sum of the ‘slight’ and ‘severe’ glomerulus numbers, the number of ‘sclerotic’ glomeruli did not affect the final classification result and could be ignored. Therefore, these rules were adopted in the evaluation group.

### 2.8. Evaluation Metrics and Statistical Analysis

The glomerular location and classification performance were evaluated using precision, recall, and F1, which are widely employed metrics for object detection. They are defined as follows:

(1) $$\mathrm{Precision}\;=\;\frac{TP}{\;TP\;+\;FP}$$

(2) $$\mathrm{Recall}\;=\;\frac{TP}{TP\;+\;FN}$$

(3) $$F_{1}\;=\;\frac{2\;\mathrm{Recall}\;\times \;\mathrm{Precision}\;}{\;\mathrm{Recall}\;+\;\mathrm{Precision}\;}$$

where *TP*, *FP*, and *FN* represent true positive, false positive, and false negative, respectively, indicating that the location is correct, the non-glomerular area is detected as the glomerulus, the glomerulus is not detected, respectively. *TN* represents true negative. The accuracy is the ratio of correctly predicting glomerular observations (*TP* + *TN*) to the total observations (*TP* + *FP* + *FN* + *TN*).

Cohen’s kappa was used to measure the agreement between the ground truth and predicted categories in the classification module. To compare the performance of this algorithm against nephropathologist at the renal level, the agreement between the AI and nephropathologist was calculated using a linear and quadratic weighted kappa, which is most useful to describe agreement when order is important. Kappa < 0 indicated no agreement, 0–0.2 slight agreement, 0.21–0.4 fair agreement, 0.4–0.6 moderate agreement, 0.61–0.8 substantial agreement, and 0.81–1 almost perfect agreement [24].

## 3. Results

### 3.1. Patients and Image Annotations

Table S1 shows the demographic characteristics and pathological diagnoses of the enrolled LN cases. The study population had a median age of 30 years (interquartile range [IQR], 24–39 years; 143 women [89.38%]). The median disease duration was 3.3 years (IQR, 0.6–7.0 years). The LN pathological diagnosis was confirmed by the three expert nephropathologists, and 50, 51, and 62 patients were categorised as classes II, III, and IV, respectively (including 1 III + V and 45 IV + V). The median serum creatinine level was 78 (IQR, 68–94) µmol/L, and 17 patients had creatinine levels >133 µmol/L. The estimated glomerular filtration rate (eGFR) of the patients was 120.43 ± 37.47 mL/(min × 1.73 m^2^), and the numbers of patients with eGFR > 90, 60–90, and 30–60 were 127, 17, and 15, respectively.

### 3.2. Glomerular Localisation Performance

The glomeruli on the low-resolution images were located through YOLOv4 and 37 WSI images (289 glomeruli) were employed for testing. Experiments showed that the localisation module could achieve a recall of 0.8308, precision of 0.9307, and F1 of 0.8779 for all types of glomeruli.

**Figure 2:**
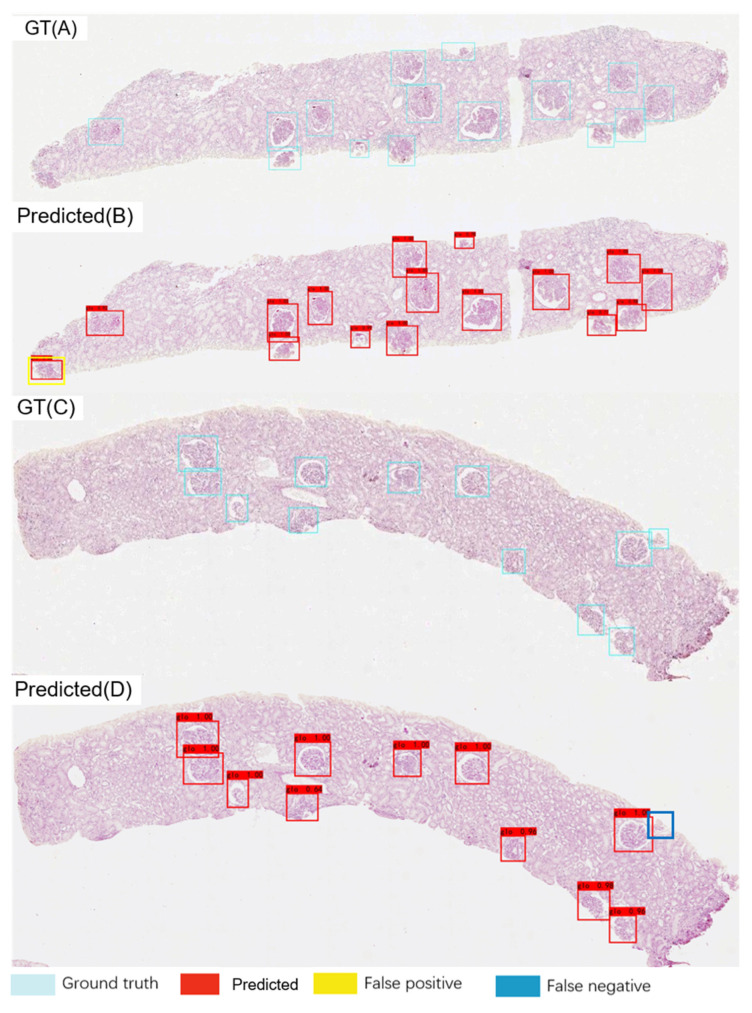
Ground truth (GT) and predicted glomerulus locations in low-resolution images. GT (**A**) and predicted (**B**) results for the same low-resolution image. The yellow box indicates a false positive result. GT (**C**) and predicted (**D**) results for another low-resolution image. The dark blue box indicates a false negative finding.

Figure 2 provides representative examples of the ground truth and glomerulus locations used in the test set. Considering the entire method, the increase in the recall rate of the localisation module is the most significant. Therefore, the localisation module confidence threshold was reduced to increase the recall to 0.9526; thus, fewer glomeruli were missing.

### 3.3. AI model Performance for Glomerular Classification at the Glomerular Level

**Figure 3:**
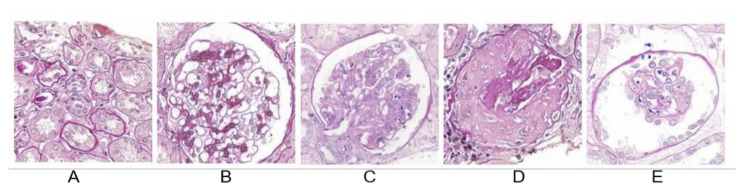
Examples of correct classification. (**A**) Regions in which the localisation module is incorrectly located (non-glomerular area). (**B**) Glomerulus with slight lesions. (**C**) Glomerulus with severe lesions. (**D**) Sclerotic glomerulus. (**E**) Incomplete glomerulus, which can be due to cutting artefacts.

In the classification module, the severity of glomerular lesions on high-resolution images was classified. VGG16 was used as the base architecture to extract the features of 1213 images of the test set and achieve glomerular classification through a fully connected layer. Figure 3 shows successful examples of multiclass classification in the test set.

**Figure 4:**
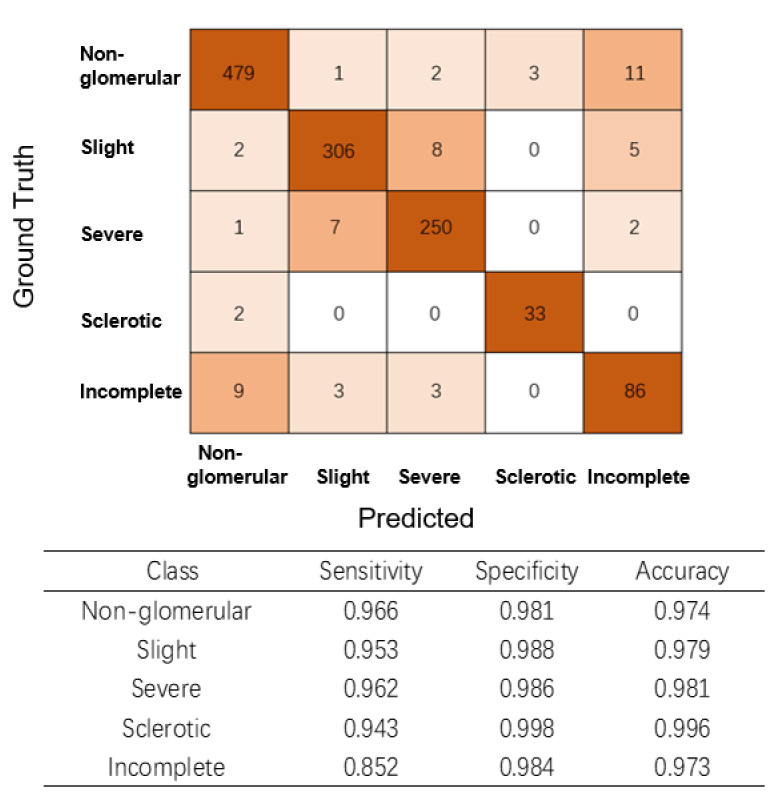
Glomerulus classification confusion matrix and performance. The ground-truth categories and labels predicted by the model are shown horizontally and vertically, respectively. The confusion matrix demonstrates the distributions of correct and incorrect classifications among different categories.

The classification module experimentally achieved an accuracy of 0.951 and Cohen’s kappa of 0.932 (95% CI: 0.915–0.949, almost perfect) in the entire test set. Moreover, as shown in Figure 4, the sensitivity and specificity values were high for ‘glomeruli with slight lesions’, ‘glomeruli with severe lesions’, and ‘sclerotic glomeruli’.

### 3.4. Glomerulus Multiclass Detection Performance

**Figure 5:**
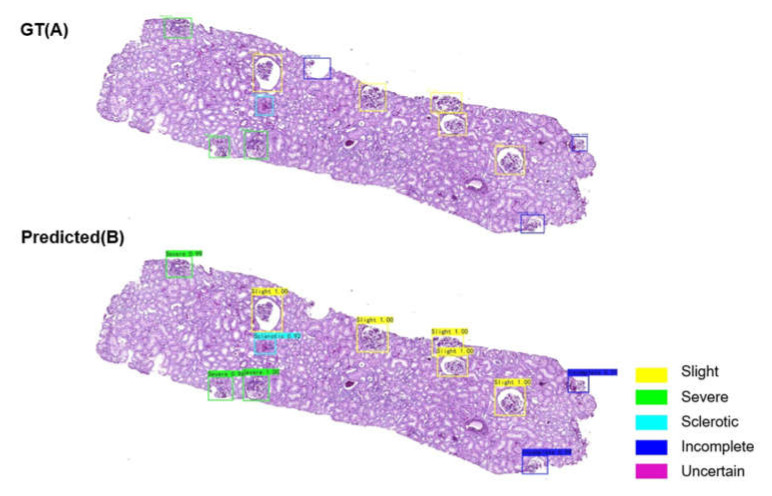
Ground truth (GT) and predicted glomerulus locations and categories in high-resolution images (WSIs). GT (**A**) and predicted (**B**) results for the same WSI. The top of the box is labelled with the category name and confidence level of the glomerulus that was detected.

To study the CNN multiclass detection performance, 37 WSIs from renal biopsies were used. According to the mean average precision of this test set, the CNN model performance was 0.807. It was not difficult for the AI-based model to distinguish between the ‘non-glomerular area’ and ‘slight’, ‘severe’, and ‘sclerotic’ glomeruli (see Figure 5 for an example). For ‘glomeruli with slight lesions’, the precision, recall, and F1 were 0.916, 0.932, and 0.924, respectively. The model performance for ‘glomeruli with severe lesions’ was similar (precision: 0.931, recall: 0.974, F1: 0.952). For ‘sclerotic glomeruli’, the precision, recall, and F1 were 0.682, 0.833, and 0.750, respectively. The performance for ‘incomplete glomerulus’ was also not good (precision: 0.739, recall: 0.773, F1: 0.756). In this test set, 13 ‘glomeruli’ were categorised into the ‘uncertain’ group, and it was found that most were incomplete or misrecognised glomeruli (11/13). Only two glomeruli required manual evaluation.

For detection of the same 349 WSIs, calculations with the AI model were performed on a GeForce GTX 1080 Ti, requiring a total of 2.04 h, with an average time of 21.09 s/WSI. The pathologists spent 16 h to 20 h on the same task with an average time of 185.67 s/WSI. Considering the average time, the AI model was approximately nine times faster than the pathologist.

### 3.5. AI Model Performance in Glomerular Classification at the Renal Level

To evaluate the model performance at the per-patient renal level, another 321 unannotated biopsy WSIs of 161 patients from the pathology database were used during the same period. The glomeruli were first located on the low-resolution images, detecting 3277 glomeruli. Because of insufficient information, 14 patients with no more than 10 complete glomeruli each were removed from subsequent analyses. Then the degree of glomerular lesions on the high-resolution images were classified using the classification module as previously described. In this dataset, 67 ‘glomeruli’ were categorised into the ‘uncertain’ group, and only 11 of these glomeruli (16.4%) had ambiguous categories (‘slight’ and ‘severe’) and needed further manual evaluation. Once all glomeruli had been classified, a kidney-level classification was generated based on a simple majority count as described before.

The AI model predictions were compared with the nephropathologist gradings and found that the AI model achieved an accuracy of 75.0–100.0% (Table 1). The linear and quadratic weighted kappa was 0.855 (95% CI: 0.795–0.916, *p* < 0.001) and 0.906 (95% CI: 0.873–0.938, *p* < 0.001) respectively which indicated almost perfect agreement. If the results of cases with only one or two ‘severe’ glomeruli were eliminated, the model accuracy for classifying classes II and III increased from 75.0% and 81.0% to 87.9% (29/33) and 89.7% (26/29), respectively.

## 4. Discussion

A deep learning-based technique was developed to locate and classify glomerular pathologies in human lupus glomerulonephritis. This objective automated method of classifying LN achieved good performance on test datasets and obtained a high agreement with nephropathologists. It will provide nephropathologists with a valuable tool to reduce their operative workload and interobserver variability by supplementing pathologist assessments.

In recent years, the successful implementation of deep learning algorithms in kidney biopsy classification has raised the hope that their use will eventually improve the reproducibility and accuracy of pathologist diagnoses. Marsh et al. developed and validated a deep learning model to quantify glomerulosclerosis in donor kidney biopsy specimens, surpassing the capacity of pathologists in a time-sensitive setting [25,26]. Ginley et al. successfully used recurrent neural network technology to classify diabetic nephropathy [24]. In immunoglobulin A nephropathy, a deep learning approach for glomerular lesion and intrinsic glomerular cell identification has also been established [27]. Glomerular hypercellularity, another lesion type, can be detected in human kidney images and classified using a CNN along with a support vector machine [28].

Different kidney diseases may share similar renal lesions; for example, class III or IV LN may present with endocapillary hypercellularity, segmental necrosis, and crescents, similar to other glomerular diseases. However, due to the complexity and diversity of renal pathologies, the methods applied in these diseases may not be translatable directly to LN image analysis. Recently, Cicalese et al. discovered that a machine learning model could successfully resolve phenotypic differences between control, non-proliferative class I/II, and proliferative class III/IV cases in both glomerular-level (26,634 segmented glomerulus images of mice) and kidney-level (87 MRL/lpr mouse kidney sections) classification tasks [29]. In contrast to their work, the authors of this current paper used human kidney biopsy specimens from needle biopsies that substantially differ in physiological and pathological features from the mouse model specimens. In contrast to the mouse model, this paper’s results can easily be applied to clinical practice and may be of great significance for LN diagnosis.

Previous studies on AI-based glomerulus location and classification and the performances of these models have been well reviewed [27,30]; however, until now, research on human renal biopsy samples has remained insufficient. The AI approaches implemented in these studies included U-Net, DenseNet, AlexNet, and support vector machine. The CNN model, a subclass of deep and machine learning, is better suited to complex tasks such as image recognition. A CNN can identify and analyse not only known histological features, but also novel and subvisual features that are not typically considered diagnostic or apparent to the human eye [31]. Thus, this approach has been increasingly applied to renal pathology [30]. In the glomerular localisation module, our detection method is based on the assessment of low-resolution images by the CNN model YOLOv4, which is simpler and faster than slider detection methods that cut WSIs into patches of high-resolution images [32,33] or the image segmentation method [27]. The performance of our model was equivalent (precision 0.931 and F1 0.878) to that of these methods.

In terms of glomerular classification, compared with the InceptionV3 model of Uchino [34], our model could directly obtain multi-category classification results, which is more convenient and less time-consuming considering the annotation process. In addition, the performance parameters of our model were high (accuracy 0.951 and Cohen’s kappa 0.932) in the whole classification test set, which seem to be higher than those obtained by Uchino. However, these values are not directly comparable because they vary with the cut-off of the output value from the model. In the later actual detection module, our method can be better matched with the localisation results, and at the renal validation level, they can also achieve good accuracy. Compared with ‘slight’ and ‘severe’ glomerular lesions, sclerotic glomeruli are less inconspicuous and, therefore, more likely to be missed during localisation, which is the main reason for the lower recall rate. Moreover, fibrotic glomeruli can be confused easily with severely disordered glomeruli, and sclerotic glomeruli are more comparable to non-glomerular tissues, so the recall rate and accuracy are not high. There are many approaches for renal image segmentation to obtain the most detailed image quantification [18,24,35,36]. This pixel-level quantification provides precise spatial and quantitative measurements of objects at different scales. However, extensive pixel-level classification requires experienced pathologists to annotate different parts of renal histological structures, which is highly time-consuming. Therefore, segmentation is not typically involved in routine pathology workflows and was, therefore, not integrated into our model. In the future, this promising approach may enable semi-quantitative phenotyping methods to be replaced with fully quantitative solutions.

This study has some limitations. First, it was based on the assessment of PAS-stained slides, and other histological stains, such as haematoxylin-eosin and Masson trichrome, immunofluorescence, and electron microscopy were not included. Thus, vital diagnostic information, such as immune complex deposition or membranous nephropathy, could not be detected and this study could not confirm whether classes III and IV LN were combined with class V. Second, various other parameters, such as tubulointerstitial inflammation or renal thrombotic microangiopathy, which is an important renal prognostic feature [37], remain to be integrated before the renal biopsy pathology can be evaluated comprehensively. Third, because of difficulties in obtaining normal or minimal mesangial LN (class I) renal tissues from needle biopsy, this study did not obtain enough cases as controls in the model. Finally, the evaluation performance of sclerotic glomeruli, which is relatively low compared with that of other pathological features, still needs to be improved. To date, challenges such as the lack of public datasets and inadequate annotations as precise annotations require extensive clinical knowledges from trained practitioners, stand in the way of clinical adoption of AI in renal histopathology [13,30]. In future work, the authors plan to expand the number of samples in the dataset and, if possible, to collaborate with other centres with experience in kidney pathology for further completion and external multicentre validation. In the meantime, the authors will investigate more advanced AI models such as the correlation learning mechanism for deep neural networks [38] and real-time image super-resolution reconstruction [39] to improve performance. Additionally, developing a deep learning approach to evaluate other histological stains such as immunofluorescence images, as performed by Giulia et al. [40], is also feasible. Furthermore, the use of AI algorithms to integrate pathological findings with clinical symptoms and laboratory test results will outperform models trained on biopsy images only and can provide more valuable information for accurate diagnosis and prognosis prediction.

In summary, this research applied deep learning to locate and classify glomerular pathologies accurately in human lupus glomerulonephritis, achieving high accuracy, high reproducibility, the incorporation of important novel and subvisual features, and high speed. With further improvement and evaluation, this system may assist pathologists by screening biopsies, providing second opinions on classification, and even presenting quantitative information on renal lesions.

## Figures and Tables

**Table 1:** AI model predictions versus nephropathologist gradings.

Nephropathologist	AI Model			Total	Accuracy (%)
	II	III	IV		
Class II	33	11	0	44	75.0
Class III	6	34	2	42	81.0
Class IV	0	0	61	61	100.0
Total	39	45	63	147	

AI, artificial intelligence.

## Data Availability

Data are not available on request due to privacy and ethical restrictions.

## Supplementary Materials

The following are available online at https://www.mdpi.com/article/10.3390/diagnostics11111983/s1, Figure S1: A screenshot of our annotation tool, the modified LabelImg, Figure S2: The network architecture of YOLOv4. Figure S3: The network architecture of VGG16, Table S1: Clinicopathological features of lupus nephritis patients at time of biopsy.
