# Supplementary material for: Deep Learning-Based Artificial Intelligence System for Automatic Assessment of Glomerular Pathological Findings in Lupus Nephritis
Source: Diagnostics (Basel). 2021 Oct 26;11(11):1983. doi: 10.3390/diagnostics11111983 (PMC8621095; doi:10.3390/diagnostics11111983)
Supplement: Supplementary file 1 [file diagnostics-11-01983-s001.zip › diagnostics-1395979-supplementary.pdf]

Supplementary Materials

Table S1: Clinicopathological features of lupus nephritis patients at time of biopsy.

Variables	Patients (n=163)
Female, n (%)	143 (89.38%)
Age, years (median, IQR)	30 (24–39)
Disease duration, years (median, IQR)	3.3 (0.6–7.0)
Age at diagnosis, years (median, IQR)	27 (21–33)
LN classification (ground truth), n (%)	
Ⅱ	50 (30.67%)
Ⅲ	51 (31.29%)
Ⅳ	62 (38.04%)
Clinical features, n (%)	
Rash	135 (82.82%)
Photosensitivity	29 (17.79%)
Alopecia	74 (45.40%)
Oral ulcerations	28 (82.82%)
Fever	75 (17.18%)
Fatigue	49 (30.06%)
Arthritis	97 (59.51%)
Vasculitis	41 (25.15%)
Leucopoenia	22 (13.50%)
Thrombocytopenia	18 (11.04%)
Hypertension	22 (13.50%)
Renal dysfunction (>133 μmol/L)	17 (10.43%)
Proteinuria, g/24 hours (median, IQR)	0.65 (0.16–2.26)
Serum creatinine, μmol/L	87.40±30.54
eGFR mL/ (min×1.73 m^2)	120.43±37.47
Serum albumin, g/dL	32.87±7.69
C3, mg/dL	50.81±26.30
C4, mg/dL	9.59±6.65
Anti-dsDNA, positive N (%)	95 (58.28%)

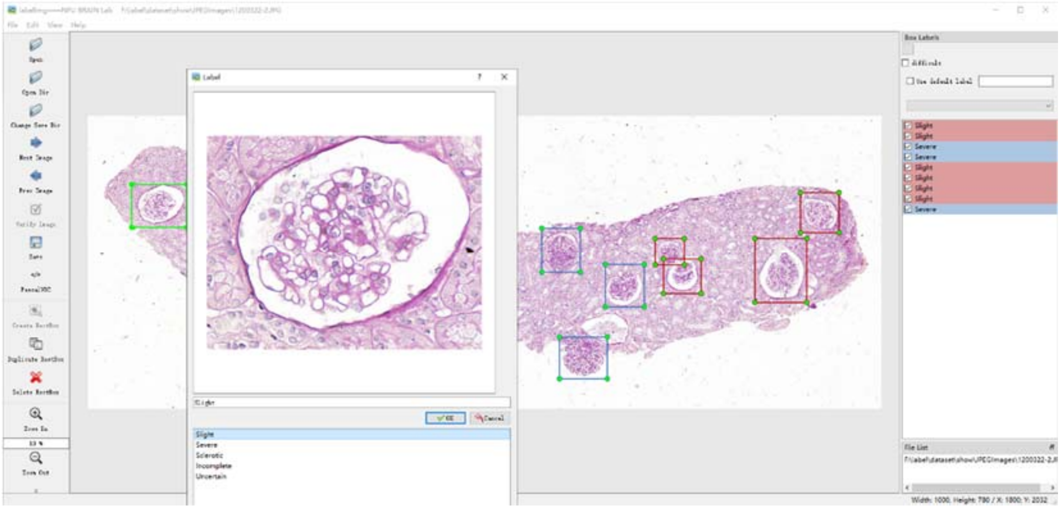

Figure S1: Screenshot of our annotation tool, the modified LabelImg.

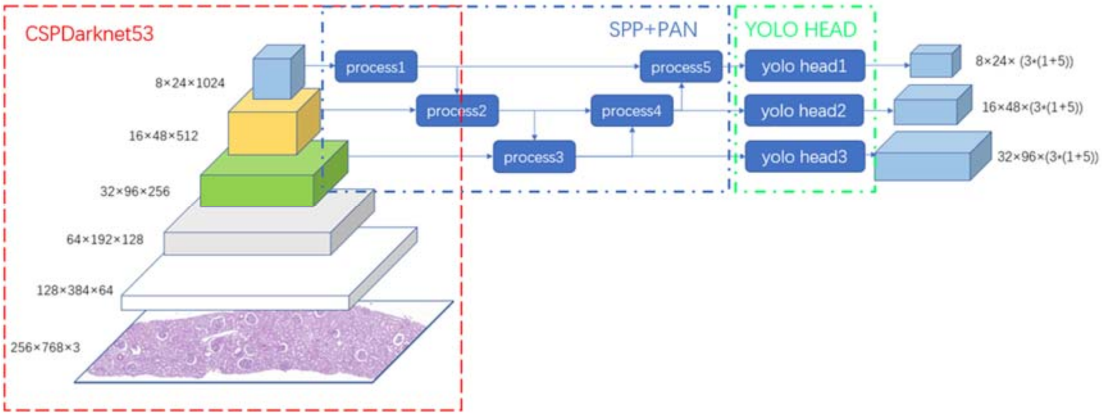

Figure S2: Network architecture of yolov4. Processes 1–5 contain operations such as convolution, upsampling, and downsampling. The SPP block is in process 1.

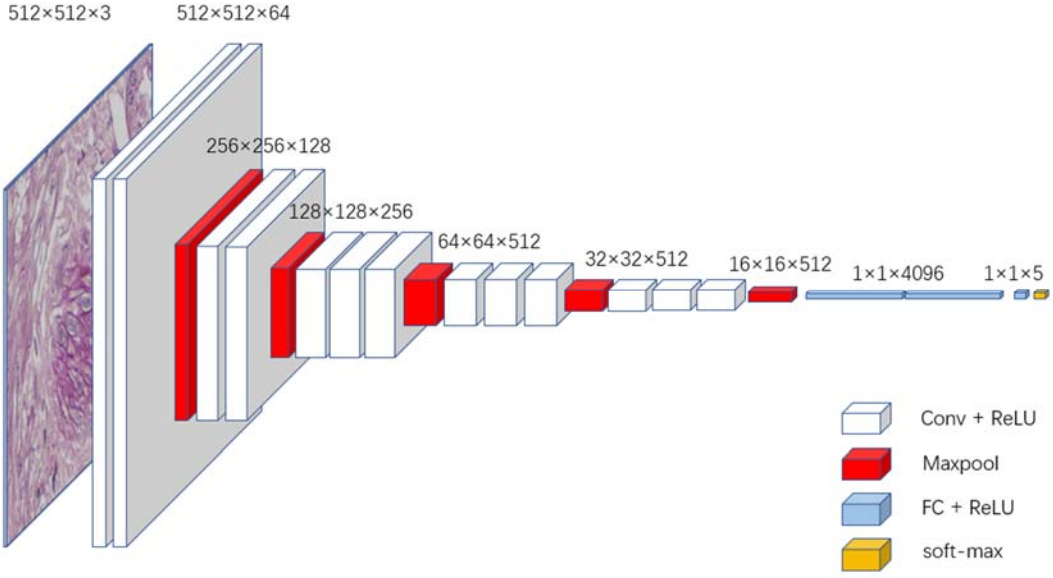

Figure S3: Network architecture of VGG16.
